# Dendritic cell CNS recruitment correlates with disease severity in EAE via CCL2 chemotaxis at the blood–brain barrier through paracellular transmigration and ERK activation

**DOI:** 10.1186/1742-2094-9-245

**Published:** 2012-10-26

**Authors:** Divya Sagar, Anne Lamontagne, Catherine A Foss, Zafar K Khan, Martin G Pomper, Pooja Jain

**Affiliations:** 1 Drexel Institute for Biotechnology and Virology Research and Department of Microbiology and Immunology, Drexel University College of Medicine, Philadelphia, PA 19129, USA; 2 Russell H. Morgan Department of Radiology and Radiological Science, Johns Hopkins Medical Institutions, Baltimore, MD 21231, USA; 3 Department of Microbiology & Immunology, Drexel Institute for Biotechnology & Virology Research, Drexel University College of Medicine, 3805 Old Easton Road, Doylestown, PA 18902, USA

**Keywords:** MCP-1, Chemokine ligand 2, Dendritic cell central nervous system trafficking, Blood–brain barrier, Near-infrared fluorescence imaging, Neuroinflammation, Brain microvascular endothelial cells

## Abstract

**Background:**

Transmigration of circulating dendritic cells (DCs) into the central nervous system (CNS) across the blood–brain barrier (BBB) has not thus far been investigated. An increase in immune cell infiltration across the BBB, uncontrolled activation and antigen presentation are influenced by chemokines. Chemokine ligand 2 (CCL2) is a potent chemoattractant known to be secreted by the BBB but has not been implicated in the recruitment of DCs specifically at the BBB.

**Methods:**

Experimental autoimmune encephalomyelitis (EAE) was induced in C57BL/6 mice by injection of MOG_35–55_ peptide and pertussis toxin intraperitoneally. Animals with increasing degree of EAE score were sacrificed and subjected to near-infrared and fluorescence imaging analysis to detect and localize the accumulation of CD11c^+^-labeled DCs with respect to CCL2 expression. To further characterize the direct effect of CCL2 in DC trafficking at the BBB, we utilized an *in vitro* BBB model consisting of human brain microvascular endothelial cells to compare migratory patterns of monocyte-derived dendritic cells, CD4^+^ and CD8^+^ T cells. Further, this model was used to image transmigration using fluorescence microcopy and to assess specific molecular signaling pathways involved in transmigration.

**Results:**

Near-infrared imaging of DC transmigration correlated with the severity of inflammation during EAE. *Ex vivo* histology confirmed the presence of CCL2 in EAE lesions, with DCs emerging from perivascular spaces. DCs exhibited more efficient transmigration than T cells in BBB model studies. These observations correlated with transwell imaging, which indicated a paracellular versus transcellular pattern of migration by DCs and T cells. Moreover, at the molecular level, CCL2 seems to facilitate DC transmigration in an ERK1/2-dependent manner.

**Conclusion:**

CNS recruitment of DCs correlates with disease severity in EAE via CCL2 chemotaxis and paracellular transmigration across the BBB, which is facilitated by ERK activation. Overall, these comprehensive studies provide a state-of-the-art view of DCs within the CNS, elucidate their path across the BBB, and highlight potential mechanisms involved in CCL2-mediated DC trafficking.

## Background

Cells of hematopoietic origin play important roles in the pathogenesis of many neurological conditions such as multiple sclerosis, Alzheimer’s disease and Parkinson’s disease, and in viral and bacterial infections of the brain such as HIV encephalitis, Japanese encephalitis, and meningitis. Immune cells constantly survey the brain microvasculature for irregularities in levels of factors signaling homeostasis. Immune responses are initiated when necessary, resulting in mobilization of resident microglial cells [1,2] within the central nervous system (CNS) and/or infiltrating peripheral cells [3,4]. A recent study of brain tissue resident dendritic cells (DCs) in steady state [5] showed that they share a similar phenotype and genotype profiling with splenic DCs. Both DC subsets have a common precursor as pre-DCs or peripheral blood DCs that are derived from the bone marrow [5,6]. Subsequently, these brain DCs were able to proliferate MOG-specific T cells in the presence of MOG peptide, suggesting its importance in antigen presentation during experimental autoimmune encephalomyelitis (EAE), a demyelinating disease model of multiple sclerosis. A quantification of cell types in spinal cord homogenates of EAE-induced mice has already proven the existence of CD11c^+^ DCs of myeloid lineage in the CNS [7]. This indicator of infiltrating DCs into the inflammatory CNS from the systemic blood circulation is indeed true, as also shown previously [8]. These cells are potent antigen-presenting cells that have the capability to accumulate in the CNS in the presence of inflammation (reviewed in [9]). In fact, CD11c-positive DCs have been shown to be sufficient to initiate this autoimmune demyelinating disorder [10]. Further, antigen presentation by myeloid DCs has been implicated in driving progression of relapsing EAE (reviewed in [11]).

High expression of chemokine ligand 2 (CCL2) has been seen in animals with a chronic relapsing etiology of EAE [12,13]. Dogan and colleagues previously demonstrated less accumulation of CD11c^+^ DCs of myeloid lineage in spinal cord homogenates of CNS CCL2^−/−^ chimeric mice induced with EAE [7]. Further, these mice have been shown to undergo less demyelination compared with wildtype controls induced with EAE. Whether this shortage in DC accumulation is a result of less mononuclear infiltration from the systemic circulation as a direct cause of CCL2 absence is uncertain. Despite being important antigen-presenting cells in EAE [10,14,15], the mechanism and degree of DC chemoattraction by CCL2 at the blood–brain barrier (BBB) interface is not known. CCL2 is known to be released by astrocytes and microvascular endothelial cells at the glia limitans, allowing chemoattraction of immune cells patrolling the BBB vasculature [16,17]. What is also known is that DCs partake in immunosurveillance and tether to the cerebral endothelium via binding of adhesion molecules present on its surface [8]. Recent intravital microscopic studies have implicated CCL2 as an important chemokine influencing immune cell adhesion to the cerebral endothelium [18,19]. CCL2 also increases BBB permeability [20], which can further permit infiltration of immune cells from the systemic circulation.

Having been investigated extensively in the context of HIV encephalitis, CCL2 has until now been shown to recruit mostly monocytes and microglia at inflammatory sites in the CNS. This chemokine diffuses paracellularly through the endothelium and attracts monocytes and macrophages to the site of arrest prior to transmigration [21]. Firm arrest of rolling monocytes on endothelial monolayers expressing E-selectin [22] and the spread of and alteration in the shape of monocytes at the endothelium [23] are triggered by CCL2. T-cell populations also possess receptors to migrate in the presence of CCL2. Further, monocytes have been implicated in pulling these T cells from the perivascular space into the parenchymal spaces. In the absence of monocytes, T cells accumulate transiently in the perivascular space, leading to delayed disease onset but also to delayed virus control in animals infected with viral encephalitis [24]. In comparison with these other immune cells, the phenomenon of DC transmigration across the BBB, however, has been only minimally explored in the context of neuroinflammation via CCL2.

Herein, we image and evaluate the ability of endogenous DCs to transmigrate across the BBB during EAE and further study their degree and mechanism of chemotaxis and transmigration in the presence of CCL2. First, we observed the correlation between severity of EAE and accumulation of DCs via near-infrared imaging analysis. Further histology confirmed that DCs were found in the CCL2-producing lesions. On the basis of *ex vivo* observations, we compared the *in vitro* kinetics of transmigration of nonactivated and activated DCs to determine CCL2-mediated chemotaxis. Both transmigration and immunofluorescence studies indicated that DCs, in comparison with T cells, were more potent responders to CCL2. We also observed that the DC transmigration pattern was primarily paracellular and dependent on ERK1/2 phosphorylation compared with that of T cells, which was transcellular and dependent on the phosphorylation of p38-mitogen-activated protein kinase (MAPK). Conclusions drawn from these comparative studies justify the development of therapies that specifically target DCs in CCL2-driven pathogenesis of neurological conditions. Results from these studies further substantiate the promise of current cell-based immunotherapies to battle diseases that circumvent body’s immune capabilities, and open new doors to generating DC-based therapies that can be directed to inflammatory lesions or tumors that express CCL2.

## Materials and methods

### Tracking dendritic cell migration in EAE mice

#### Generation of EAE in mice

All animal procedures were approved and carried out under full compliance with the Johns Hopkins University Animal Care and Use Committee guidelines. Six-week-old to 8-week-old female C57BL/6 mice (Jackson Laboratory, Bar Harbor, ME, USA) were acclimated, inoculated, conditioned, and scored for EAE according to [25]. Briefly, each mouse was injected (subcutaneously) with 100 μl of 2 mg/ml MOG_35–55_ peptide and received 250 ng pertussis toxin intraperitoneally at inoculation and 2 days later. All mice were scored to confirm EAE induction, from which three were picked based on increasing degree of disease severity and were injected with labeled antibodies on day 14 post immunization and imaged on day 16.

#### Labeling of antibodies

Samples of 60 μg anti-CD3 antibody, anti-CD11c antibody and anti-MBP (anti-myelin basic protein) antibody (Abcam, Cambridge, MA, USA) were labeled with either IRDye800CW-NHS ester (anti-CD11c) or IRDye680-NHS ester (25 ng specified dye; Li-Cor Biosciences, Lincoln, NE, USA) in 100 μl PBS. Reactions were allowed to sit at room temperature for 12 minutes before the reactants were loaded onto a PBS-conditioned Sephadex G-25 size-exclusion column (Invitrogen, Carlsbad, MA, USA) and spun at 2,600×*g* for 2 minutes. Purity was confirmed by Gelman TLC in ACD buffer (Sigma, St. Louis, MO, USA). Each antibody was ≥95% pure after purification.

#### Near-infrared fluorescence imaging

C57BL/6 mice with EAE were injected (intraperitoneally) with indicated fluorescently labeled probes on day 14 post MOG inoculation. Mouse 1 was injected with 15 μg purified anti-CD11c Ab-IRDye800 only. Mouse 2 was co-injected with 20 μg anti-CD11c Ab-IRDye800 and 15 μg anti-CD3 Ab-IRDye680. Mouse 3 was co-injected with 15 μg anti-CD11c Ab-IRDye800 and 15 μg anti-MBP Ab-IRDye680. Mice were imaged *ex vivo* 48 hours later using a Pearl Impulse imager employing fixed 710 nm and 800 nm bandpass emission filters (Li-Cor Biosciences). The mice were first sacrificed by cervical dislocation and trimmed down to the spine and skull to allow high-contrast visualization of fluorescence from these tissues. The brains and spines were frozen and sectioned to 20 μm using a Microm HM 550 cryotome (Thermo Scientific, Waltham, MA, USA).

#### Ex vivo *microscopy*

Frozen sections were either left unfixed or fixed with 10% formalin for 20 minutes at room temperature prior to two brief washes with PBS. Unfixed sections were probed with anti-CD31-PE antibody (1:67; Abcam), anti-CD68 antibody (1:83; Abcam) and anti-CCL2 antibody (1:33; Abcam) for 1 hour in 10% fetal bovine serum in PBS to delineate the presence of angiovasculature and chemokine, respectively. Fixed sections were probed only with anti-CCL2 antibody. Goat anti-rabbit-AlexaFluor488 (1:250; Invitrogen) and sheep anti-rat conjugated to fluorescein (1:250; Abcam) secondary antibodies were then added to detect the anti-CCL2 primary antibody for 30 minutes at room temperature. Lastly, Hoechst 33342 dye was introduced for 90 seconds prior to two brief washes. The slides were then mounted in Faramount Aqueous Mounting Media (Dako North America, Carpinteria, CA, USA) with a glass coverslip and viewed 30 minutes later using a Nikon 80i upright epifluorescence microscope equipped with a Nikon DS-Qi1Mc darkfield CCD camera and excited by a Nikon Intensilight C-HGFI lamp (Nikon, Melville, NY, USA). All images were recorded and processed using Nikon Imaging Software Elements (Nikon, Melville, NY, USA).

### Construction of blood–brain barrier models

To create a cellular monolayer barrier, polyethylene tetraphthalte transwell inserts with a pore size of 8.0 μm were coated with matrigel matrix (BD Biosciences, San Diego, CA, USA), a model basement membrane. Primary human brain microvascular endothelial cells were then cultured to 100% confluency on the upper side of the membrane inserts. For the three-cell BBB model, after the formation of the monolayer, inserts were transferred to a six-well plate that contained primary human fetal astrocytes (ScienCell Laboratories, Carlsbad, CA, USA) and retinoic acid-differentiated post-mitotic neuronal (NT2) cells as described previously [26]. The inserts were kept in close juxtaposition to the surface of the chamber of the six-well plate, allowing intimate contact with the astrocytes and neurons. In both cases, the integrity of the BBB formed was verified by microscopic observation as well as through trans-endothelial electrical resistance (TEER) determination by ENDOHM-6 (World Precision Instruments, Inc., Sarasota, FL, USA) at various time intervals (24, 36 and 48 hours). The permeability of the BBB was determined by treating transwells with 0.3% ethanol overnight. Inserts were then transferred to another well with fresh medium and 150 μl of a 1 mg/ml solution of FITC-dextran (Sigma-Aldrich, St Louis, MO, USA) were added to each insert for 12 hours. Medium from the bottom chamber was collected and florescence was measured using a multiwell plate reader with extinction at 480 nm and emission at 530 nm.

### Determination of cell purity

For transmigration assays, primary DCs and T cells (CD4 and CD8) were utilized. Highly purified monocyte-derived dendritic cells (MDDCs) were obtained from the buffy coat of healthy blood donors as described elsewhere [27]. Briefly, peripheral blood mononuclear cells were isolated from heparinized blood by Ficoll-Paque Plus (Amersham Biosciences, Uppsala, Sweden) density gradient centrifugation. Monocytes were allowed to adhere to the bottom of six-well plates, and nonadherent peripheral blood lymphocytes (PBLs) were separated by washing. The adherent monocytes were cultured in 1% normal human plasma (Sigma-Aldrich) in the presence of rhGM-CSF (100 IU/ml; PeproTech, Rocky Hill, NJ, USA) and rhIL-4 (300 IU/ml; PeproTech) for 5 days at 37°C and 5% CO_2_. Cells were provided with fresh cytokines every other day. MDDCs were identified as a Lin-1^–^/HLA-DR^+^ population by flow cytometry. PBLs were subjected to the EasySep human CD4^+^ or CD8^+^ T-cell enrichment kit (StemCell Technologies, Vancouver, BC, Canada). The purities of the CD4^+^ and CD8^+^ fractions were found to be >90% by fluorescence-activated cell sorting analyses using FITC-conjugated CD4 or CD8 antibody (eBioscience, San Diego, CA, USA). MDDCs and PBLs were further activated for 24 hours with lipopolysaccharide and phytohemagglutinin, respectively (1 μg/ml; Sigma). Activation for MDDCs was confirmed by CD86 and for T cells by CD69 marker. BD FACS Calibur was used for fluorescence acquisition using CellQuest Pro Software and data were analyzed with FlowJo software (v. 8.8.6; Tree Star, Ashland, OR, USA).

### Immune cell transmigration

Both nonactivated and activated primary cells were used in duplicate and performed at three independent settings using immune cells purified from three different donors. One million cells were transferred to the upper chamber of polyethylene tetraphthalte transwells in the monolayer or three-cell BBB model and allowed to transmigrate for 48 hours. At 24-hour intervals, transmigrated cells from the bottom chamber were removed and counted by trypan blue exclusion. In addition, cells were separately labeled with FITC-dextran (1 mg/ml) and then added to the upper chamber of the *in vitro* BBB. At 24-hour intervals, transmigrated cells were used to measure the florescence as described above. Where indicated, CCL2 (R&D Systems, Minneapolis, MN, USA) was added to the lower chamber at varying concentrations (50, 100, and 200 ng/ml) at the same time as immune cells were added to the upper chamber. The entire data were statistically analyzed to determine the average of duplicate samples or the median of replicate experiments. Comparisons were made between the control and experimental samples and a *P* ≤0.05 value by the Student’s *t* test was considered significant.

### *In vitro* imaging of the transwells

*In vitro* imaging was performed on an immortalized human cerebral microvascular endothelial cell line (hCMEC/D3) obtained from Dr Pierre-Olivier Courard (Institut Cochin, Paris, France). The cells were maintained as described previously [28] and grown on collagen-treated 3-μm inserts in a 24-well format (BD Biosciences). Confluent monolayers were confirmed both visually and by TEER measurement. Nonactivated immune cells (2×10^5^ MDDCs or PBLs) were labeled with 4',6-diamidino-2-phenylindole (DAPI) and added to the upper chamber of the transwell while medium without or with CCL2 (100 ng/ml) was added to the lower chamber. At various times post addition (10 and 30 minutes, 2, 4, and 24 hours), membranes were washed and fixed with 4% paraformaldehyde for 10 minutes followed by blocking and probing with anti-ICAM-1 (Abcam) and anti-caveolin-1 (Cell Signaling Technology, Danvers, MA, USA) monoclonal antibodies in succession, for 1 hour. Further, anti-mouse-AlexaFluor488 (Invitrogen) and anti-rabbit-TRITC (Jackson ImmunoResearch, West Grove, PA, USA) secondary antibodies were used, followed by mounting with VectaShield mounting medium (Vector Labs, Burlingame, CA, USA). A series of 20× and 100× z-stack images were collected using the Olympus IX81 inverted microscope and were analyzed using 3I Slidebook software (Olympus, Center Valley, PA, USA).

### Effect of p38 and ERK signaling on cellular transmigration

Both nonactivated and activated MDDCs and PBLs were lysed with M-PER Mammalian Protein Extraction Reagent (Thermo-Pierce, Rockford, IL, USA) and protein concentrations were determined by the Bradford assay. Equal amounts of proteins for each sample were resolved on 12% SDS-PAGE and blotted onto the PVDF membranes. Membranes were blocked with Odyssey blocking buffer (Li-Cor Biosciences) and probed with antibodies for p38 and phosphorylated p38-MAPK (Invitrogen) as well as ERK and phosphorylated ERK1/2 (Cell Signaling Technology), all at 1:500 dilution followed by appropriately conjugated secondary antibodies. Signals were detected using the Odyssey infrared imager (Li-Cor Biosciences). In order to test the effect of p38 and ERK inhibition, transmigration assays were further set-up in 24-well format with hCMEC/D3 cells in the absence or presence of 100 ng/ml CCL2 (as described above). Nonactivated and activated MDDCs and PBLs either were left untreated or were pretreated with inhibitors for p38 (SB203580; Invitrogen) and ERK1/2 (U0126; Cell Signaling Technology) in varying doses (25, 50, and 100 μM) for 1 hour. Cells were washed and added to the transwells for 24 hours. At the end of incubation, cells were collected from the lower chamber and were counted.

## Results

### Accumulation of DCs correlates with disease severity in mice with EAE

**Figure 1:**
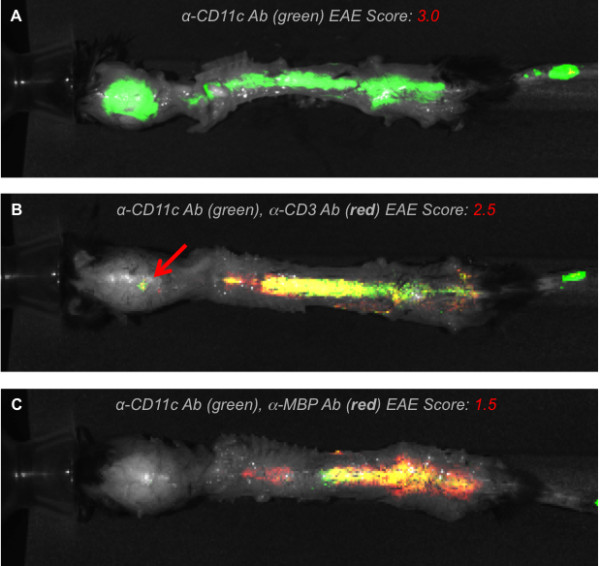
**Distribution of CD11c**^**+ **^**dendritic cells in d16 experimental autoimmune encephalomyelitis mice.*** Ex vivo* near-infrared fluorescence imaging of d16 experimental autoimmune encephalomyelitis (EAE) mice showing the distribution of CD11c^+^ dendritic cells (DCs) in the context of CD3^+^ T cells and myelin basic protein (MBP). (**A**) Anti-CD11c antibody only (green) signal from DCs in a mouse with severe degree of EAE. (**B**) Mouse with moderate EAE score shows signal from both CD11c^+^ DCs (green) and CD3^+^ T cells (red) along the thoracic and lumbar spine. (**C**) Mouse exhibiting mild EAE shows a high degree of co-localization between CD11c^+^ DCs and MBP signal.

MOG peptide was injected into C57BL/6 mice to induce EAE within 14 days. As expected, these mice developed differing degrees of disease severity ranging from 3.0 (severe) to 2.5 (moderate) to 1.5 (mild) (Figure 1), providing a valid range to observe DC migration pattern with increased inflammation. To visualize infiltration of DCs into areas of demyelination, *ex vivo* near-infrared imaging was undertaken in these mice by co-injection of fluorescently labeled probes to track endogenous DCs and T cells noninvasively. The probe intensities for all mice were scaled to the same exposure time. The anti-CD11c antibody (DC) distribution was consistently displayed in the brains and spinal cords of all three mice, reflecting both increased DC accumulation as well as some nonspecific inflammatory accumulation due to whole IgG binding correlating with increased disease severity (Figure 1A,B,C). Figure 1A shows the extent of CD11c^+^ DC infiltration (green) throughout the length of the spine and into the brain, as reflected by the intensity and distribution of the probe. This mouse had the most severe phenotype, consisting of complete rear leg paralysis with partial front leg paralysis. Mouse 2 in Figure 1B again shows CD11c^+^ DCs in green throughout the thoracic and lumbar spine and it co-localizes almost entirely with the CD3 T-cell signal seen in the same tissue (red), including a discrete lesion seen in the brain (arrow). Mouse 2 had a less severe EAE phenotype consisting mainly of hind leg paralysis with no front leg paralysis. Mouse 3, shown in Figure 1C, had a mild EAE score consisting of hind limb dragging only and this mouse displayed the least amount of CD11c^+^ DC probe (green), which co-localized almost completely (yellow) with the uptake of anti-MBP antibody (red). The MBP probe shows sites of active lesions where DCs could pick up antigen for presentation in draining lymph nodes. Overall, these results suggest that the trafficking pattern of DCs into CNS lesions follows the degree of inflammation.

### Dendritic cells co-localized with myelin basic protein and concentrated within perivasculature tissue near CCL2-producing lesions

**Figure 2:**
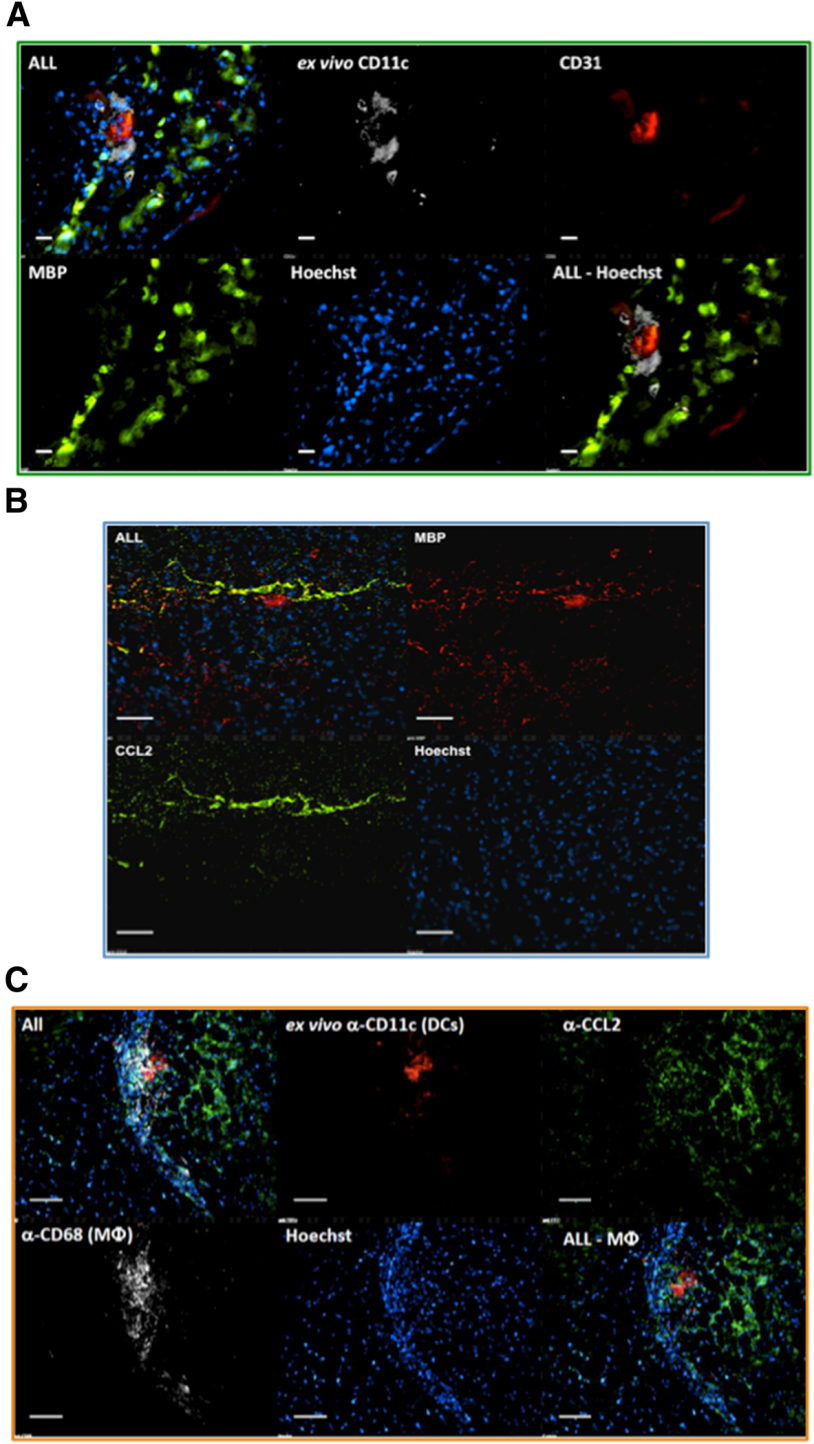
**Dendritic cells are largely perivascular indicating transmigration to CCL2 producing experimental autoimmune encephalomyelitis lesion areas.** (**A**) Brain from Mouse 3 (Figure 1) stained with anti-CD31 and/or anti-chemokine ligand 2 (anti-CCL2) antibody and Hoechst 33342 dye. In all fields, CCL2 staining (green) was closely associated with myelin basic protein (MBP) staining (red). (**B**) Brain from Mouse 1 probed *in vitro* with anti-MBP antibody (lesions), anti-CD31 antibody (blood vessel endothelium) and Hoechst 33342 nuclear dye. The anti-CD11c signal represents *ex vivo* staining. In all fields, CD11c^+^ staining (white) was confined to perivascular tissue and did not extend beyond four or five cell layers, which is typical of this model [25]. (**C**) Dendritic cells are found in proximity to CCL2 in the central nervous system of mice with experimental autoimmune encephalomyelitis **(**EAE). Brain from Mouse 3 (Figure 1) stained with additional anti-CCL2 antibody (green), anti-CD68 (white) and Hoechst 33342 dye (blue). The anti-CD11c signal (red) represents *ex vivo* staining. CD11c^+^ dendritic cells (DCs) were always in clusters and were associated with depositions of CCL2. Data represent images from multiple tissue sections. Bar = 50 μm.

To attribute the degree of CNS inflammation to the accumulation of DCs, tissue sections from the mice in Figure 1 were labeled for the important inflammatory marker CCL2. The juxtaposition of DCs with respect to CCL2 and to the site of the lesions was studied. We sought to confirm the localization of CD11c^+^ DCs in the context of vascular versus parenchymal tissue as well as their distribution in the presence of CCL2 cytokine and anti-MBP antibody delineated axonal lesions. Our results confirmed that CD11c^+^ DCs were clustered around the blood vessel that was adjacent to the expanding field of lesions (Figure 2A). We then determined whether such a CCL2 gradient was associated with demyelinating lesions in the EAE model used in these experiments. Lesioned cerebellum containing fluorescent *ex vivo* anti-MBP antibody was subsequently fixed and probed with anti-CCL2 antibody and Hoechst nuclear stain. Figure 2B shows a staining pattern for CCL2 that is closely associated with and branching out from the MBP lesion pattern displayed in red. The CCL2 seen here may have been generated by reactive astrocytes or perivascular macrophages [29] closely associated with nascent lesions, as in Figure 2A. The presence of DCs in areas expressing CCL2 (Figure 2C) suggests chemoattraction of these cells towards sites of lesion expressing CCL2. Further, anti-CD68 stained macrophages seen near CCL2-producing areas served as a positive control. Control mice without EAE did not demonstrate any infiltration of DCs.

### A three-cell model exhibits more resistance than a one-cell BBB model

**Figure 3:**
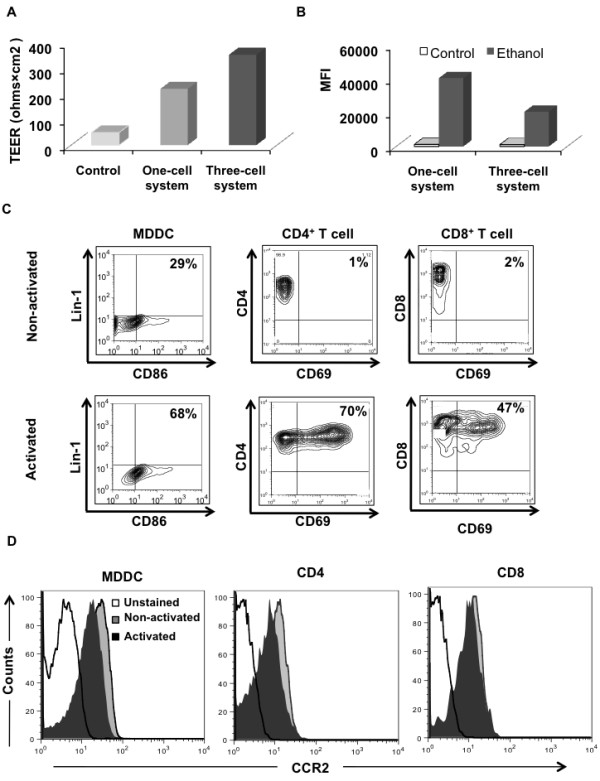
**Immature monocyte-derived dendritic cells express higher CCR2 levels compared with CD4**^**+ **^**and CD8**^**+ **^**T cells.** (**A**) Transendothelial electrical resistance measured from both the one-cell and three-cell systems. (**B**) Blood–brain barrier (BBB) permeability following ethanol treatment. Data are representative of at least two independent experiments. (**C**) Nonactivated (top panel) or activated (bottom panel) cells were surfaced-stained with appropriate antibodies as described and analyzed by flow cytometry. Thirty thousand events were gated to include the Lin-1^–^, CD4^+^, and CD8^+^ populations for monocyte-derived dendritic cells (MDDCs) and T cells, respectively, and were then analyzed for the specific activation markers (that is, CD86 for MDDCs and CD69 for T cells) by flow cytometry. Numbers represent the percent positive population. (**D**) Nonactivated and activated MDDCs and peripheral blood lymphocyte (PBLs) were gated for their respective markers and then analyzed for the surface expression of chemokine ligand 2 receptor (CCR2) by flow cytometry. Phenotyping analyses are representative of cells obtained from at least three donors.

Endothelial monolayers alone and in conjunction with astrocytes and neurons are accepted means to investigate cellular interaction with the BBB *in vitro*. We therefore utilized both systems in our studies to compare the transmigratory potential of MDDCs and T cells in their native and activated states as well as in response to CCL2. To ensure that the BBB models we used to study immune cell transmigration were not permeable, we determined the transendothelial resistance over time after seeding endothelial cells. A TEER value >200 Ω×cm^2^ was considered a criterion for an established and intact *in vitro* BBB. At 48 hours, one-cell and three-cell systems exhibited TEER values of 220 and 353 Ω×cm^2^, respectively (Figure 3A), suggesting the formation of a tight barrier. We then evaluated the permeability of the BBB by forced breaching in the presence of 0.3% ethanol. We added FITC-dextran to the upper chamber and, as expected, observed low permeability in both one-cell and three-cell models (mean fluorescence intensity (MFI) 1,295 and 1,161, respectively); however, upon ethanol exposure we detected a huge influx of FITC-dextran in both models (one-cell, MFI 40,633; three-cell, MFI 20,633) (Figure 3B).

### MDDCs express higher levels of chemokine ligand 2 receptor in comparison with CD4^+^ and CD8^+^ T cells

To assess purity and the activation status, we performed phenotypic analyses on MDDCs, CD4^+^ cells and CD8^+^ cells. Figure 3C clearly shows that the populations of MDDCs and CD4^+^ and CD8^+^ T cells were pure and that activation markers are expressed (lin1^–^CD86^+^, CD4^+^CD69^+^ and CD8^+^CD69^+^, respectively). To ensure an effect of CCL2 on immune cells, we examined the expression of its cognate receptor, CCR2, on these cells. Interestingly, we observed increased expression of CCR2 on the surface of nonactivated and activated MDDCs as compared with CD4^+^ or CD8^+^ T cells (Figure 3D).

### MDDCs are potent responders to CCL2-driven transmigration across the endothelial monolayer

**Figure 4:**
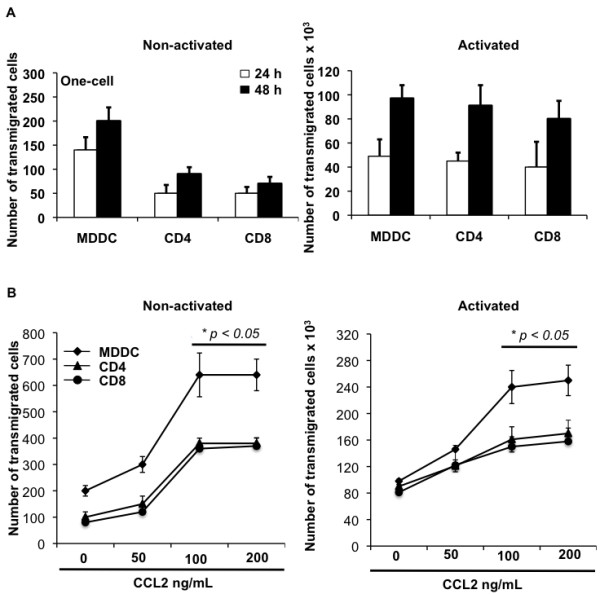
**CCL2 stimulates activated and nonactivated monocyte-derived dendritic cells and T cells to transmigrate.** Chemokine ligand 2 (CCL2) stimulates activated and nonactivated monocyte-derived dendritic cells (MDDCs) and T cells to transmigrate through the blood–brain barrier (BBB) monolayer. (**A**) One million immune cells were added to the upper chamber of the one-cell BBB model and collected after 24 and 48 hours from the lower chamber and counted. Data shown are the median of three replicate samples from three different donors ± standard deviation. (**B**) Nonactivated and activated MDDCs and T cells were added to the upper chamber of the one-cell model containing CCL2 in the lower chamber. Cells were collected 48 hours post migration and counted. Data are representative of at least two independent experiments each performed in duplicate. Statistical significance (*P ≤0.05) between the control (no CCL2) and each CCL2-treated sample was determined by Student’s *t* test.

Upon observing the presence of DCs at the site of CCL2-producing demyelinating lesions (Figure 2), we queried whether this perivascular presence was a result of the direct chemoattraction of MDDCs by CCL2. Using the one-cell BBB model, we measured the transmigration of primary MDDCs, CD4^+^ and CD8^+^ T cells in the absence and presence of CCL2. The migratory potential of nonactivated MDDCs was much greater than T cells in the absence of CCL2 (Figure 4A, left); however, upon activation, the cells were all able to transmigrate efficiently (Figure 4A, right). Both nonactivated and activated cells responded in a dose-dependent manner in the presence of CCL2 (Figure 4B). In addition, the number of transmigrated cells was significantly higher (*P* ≤0.05) at both 100 ng/ml and 200 ng/ml doses of CCL2. Overall, DCs demonstrated the maximal response to CCL2, which could be attributed to the comparatively higher expression of CCR2 on these cells as observed in Figure 3D where we have quantified the geometric MFI of CCR2 levels on immature MDDCs (geometric MFI 15.8), CD4^+^ T cells (geometric MFI 7.41) and CD8^+^ T cells (geometric MFI 8.09) after subtracting background values. Increased CCL2-mediated transmigration by DCs could also be due to the differential mechanism of transmigration utilized by DCs and T cells.

### MDDCs transmigrating across a three-cell model showed a reduced yet similar response to CCL2

**Figure 5:**
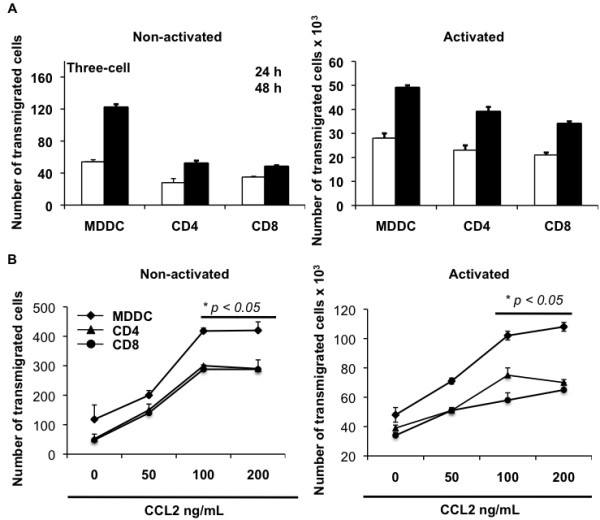
**Monocyte-derived dendritic cells and T cells transmigrate in a three-cell blood–brain barrier model.** Monocyte-derived dendritic cells and T cells transmigrate in response to chemokine ligand 2 (CCL2) in a three-cell blood–brain barrier (BBB) model. (**A**) One million immune cells were added to the upper chamber of the three-cell BBB model and collected at 24 and 48 hours from the lower chamber. Data shown are the median of three replicate samples from three different donors ± standard deviation. (**B**) Nonactivated and activated monocyte-derived dendritic cells (MDDCs) and T cells were added to the upper chamber of the three-cell model containing CCL2 in the lower chamber. Cells were collected at 48 hours post migration and counted. These data are representative of at least two independent experiments each performed in duplicate. Statistical significance (*P ≤0.05) between the control (no CCL2) and each CCL2-treated sample was determined by Student’s *t* test.

A typical neurovascular unit contains astrocytes and neurons in close juxtaposition with endothelial cells, so we compared our results from Figure 4 with an established three-cell BBB model. The overall number of transmigrating cells was much lower than in the one-cell model, yet both demonstrated a similar pattern of transmigration. In Figure 5 we again show that activated immune cells transmigrate more efficiently than nonactivated cells and that CCL2 enhances the migratory potential (Figure 5, bottom). These results suggest that cellular trafficking across the BBB primarily depends upon the interaction of immune cells and endothelial cells and justified our use of the one-cell BBB model in subsequent imaging and mechanistic studies.

### MDDCs transmigrate paracellularly whereas T cells transmigrate transcellularly

**Figure 6:**
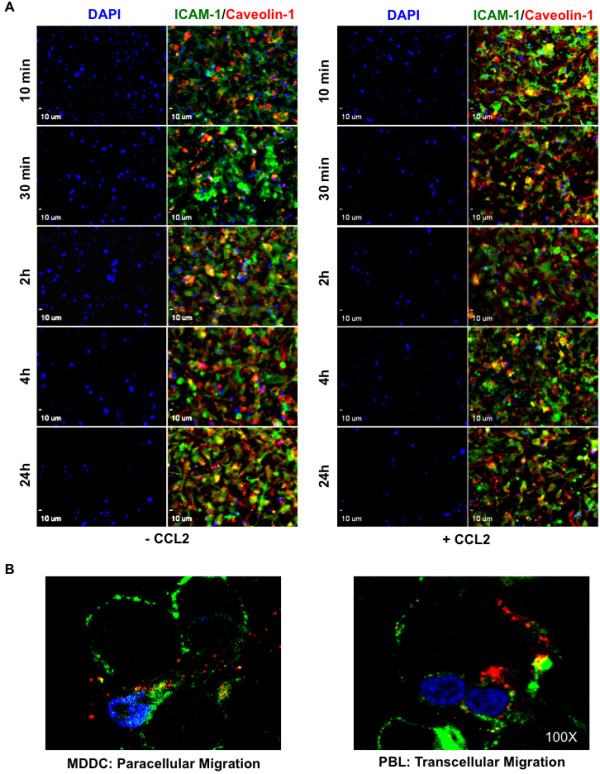
***In vitro *kinetics and movement of monocyte-derived dendritic cells across the endothelium responding to CCL2.** (**A**) Human brain microvascular cells (hCMEC/D3) were grown to confluence on collagen-coated polyethylene tetraphthalte membrane transwell inserts with 3 μm pores. Monocyte-derived dendritic cells (MDDCs) (2×10^5^) were labeled with 4',6-diamidino-2-phenylindole (DAPI) and added to the upper chamber while medium with or without 100 ng/ml CCL2 was added to the lower chamber. At indicated times, transwells were washed, stained for ICAM-1 (green) or caveolin-1 (red) and viewed at 20× magnification. (**B**) MDDCs and peripheral blood lymphocyte (PBLs) were labeled with DAPI and added to the upper chamber of the transwell. At 30 minutes after addition of the immune cells, the transwells were washed, stained for ICAM-1 (green) or caveolin-1 (red) and viewed at 100× magnification to determine paracellular versus transcellular migration patterns. (See also Additional file 1: Movie S1 and Additional file 2: Movie S2.).

We extended the use of the BBB monolayer model to visualize immune cell–endothelium interaction during transmigration in the absence and presence of CCL2 by *in vitro* imaging techniques. As these experiments required extensive standardization to capture real-time events on transwell membranes, we utilized the well-characterized hCMEC/D3 cell line [28]. For these experiments, we pre-labeled MDDCs with DAPI before adding to the upper chamber while the lower chamber contained medium with or without CCL2. At indicated times, transwells were fixed and stained for ICAM-1 (green) or caveolin-1 (red), and viewed at 20× magnification. Figure 6A (left) shows minimal chemotaxis in the absence of CCL2, as evidenced by the relative stability of DAPI staining up to 24 hours. Relatively few changes were seen in ICAM-1 and caveolin-1 staining, indicating monolayer integrity over a 24-hour period. On the other hand, the monolayer showed decreased ICAM-1 and caveolin-1 staining and the appearance of large gaps within 30 minutes of adding CCL2 (Figure 6A, right). Accordingly, the majority of 2×10^5^ DCs added transmigrated across the BBB as early as 30 minutes post CCL2 addition. To better understand differences in transmigration of DCs and PBLs, we characterized the interaction of immune cells with the endothelium to determine differential routes of transmigration. A careful observation of 100× images suggested that MDDCs move paracellularly (Figure 6B, left), whereas PBLs traveled through the endothelial cells in a manner consistent with transcellular migration (Figure 6B, right). ICAM-1 (green) and caveolin-1 (red) appeared to be involved in this process, because they could be seen to surround the DAPI-labeled leukocytes, encapsulating them as they bypassed the endothelial cells. The impact of these images is perhaps more clearly demonstrated by viewing the movies created from the planes of the z-stacked images (Additional file 1: Movie S1 and Additional file 2: Movie S2).

### CCL2-induced upregulation of ERK1/2 regulates MDDC transmigration, while p38-MAPK influences T-cell transmigration across the BBB

**Figure 7:**
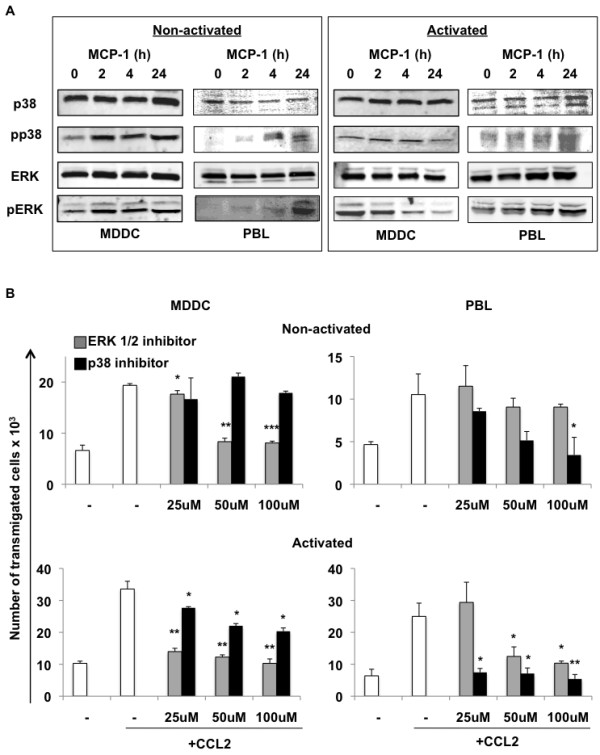
**ERK1/2 phosphorylation in nonactivated monocyte-derived dendritic cells and ERK1/2 importance in CCL2-driven MDDC transmigration.** (**A**) Nonactivated (left panel) and activated (right panel) monocyte-derived dendritic cells (MDDCs) and peripheral blood lymphocyte (PBLs) were either untreated or treated with 100 ng/ml chemokine ligand 2 (CCL2). Expression of p38-MAPK and ERK1/2 and their phosphorylated forms was determined by western blotting. (**B**) Nonactivated (top panel) and activated (bottom panel) MDDCs and PBLs (2×10^5^ cells) were left untreated or treated with p38 (SB203580) or ERK1/2 (U0126) inhibitors at the indicated doses. Transmigration assays in the presence or absence of CCL2 were performed and cells were counted at 24 hours. Data are representative of at least two independent experiments each performed in duplicate. Statistical significance (*P ≤0.05, **P ≤0.005, ***P ≤0.0005) between the control (no inhibitor) and each inhibitor-treated sample in the presence of CCL2 was determined by Student’s *t* test.

To analyze intracellular molecular events taking place in response to CCL2 stimulation of MDDCs and T cells, we studied two important signaling molecules from the perspective of cell migration, p38-MAPK and ERK1/2. Expression of these signaling proteins and their phosphorylated forms were detected in untreated cells and in cells treated with CCL2 for 2, 4 and 24 hours. In response to CCL2 stimulation, the levels of p38 and ERK1/2 did not change in nonactivated or activated MDDCs and PBLs (Figure 7A). As early as 2 hours following CCL2 exposure, however, both nonactivated MDDCs and PBLs showed upregulation of phosphorylated p38 (Figure 7A, left). Activated PBLs showed steady phosphorylated p38 expression, while MDDCs seem to lose expression of this molecule upon activation (Figure 7A, right). Both nonactivated MDDCs and PBLs showed strong activation of ERK1/2 (phosphorylated ERK), with a much earlier upregulation seen in MDDCs (Figure 7A, left). Interestingly, activated cells (Figure 7A, right) showed similar and potent expression of phosphorylated ERK1/2, suggesting a mechanistic explanation as to why activated cells transmigrated more efficiently (Figures 4 and 5). Of note, activated MDDCs lost expression of phosphorylated ERK over time, suggesting that continuous activation of this pathway may not be needed once cells have transmigrated.

To further delineate the role of these signaling molecules in CCL2-driven transmigration, we inhibited p38-MAPK and ERK1/2 activation with commercial inhibitors (SB203580 and U0126, respectively) at indicated doses (Figure 7B). SB203580 is a class of pyridinyl imidazoles that specifically inhibits activation of MAPKAPK-2 by p38 MAPK and subsequent phosphorylation of HSP27 [30], while U0126 very selectively inhibits the kinase activity of MEK1/2 thus preventing the activation of MAPK p42 and MAPK p44, which are encoded by the ERK1 and ERK2 genes respectively [31]. After 24-hour CCL2 exposure, U0126-treated nonactivated MDDCs added to the transwells showed a striking dose-dependent decrease in transmigration, whereas no significant inhibition was achieved with SB203580, suggesting the importance of ERK1/2 signaling in DC transmigration (Figure 7B, top left). Conversely, ERK1/2 inhibition showed no effect in transmigration of nonactivated PBLs yet blocking p38 phosphorylation showed a dose-dependent decrease, with the most significant decrease at 100 μM SB203580 (Figure 7B, top right). While activated p38 was expressed in both cell types (Figure 7A), we show that it has a much greater influence on T-cell transmigration (Figure 7B, right). However, ERK activation occurs rapidly in MDDCs following CCL2 exposure and significantly affects transmigration, as evidenced by the drop in transmigration following U0126 treatment (Figure 7B, bottom left).

## Discussion

Brain microvascular endothelial cells along with their neighboring components – astrocytes, pericytes, perivascular microglia, and neurons – contribute to a unique crosstalk that is crucial for the formation and maintenance of a functional BBB. CCL2 is the principal chemokine secreted at the BBB by astrocytes, endothelial cells, and neurons during inflammatory insult [32] and leads to immune cell recruitment to areas of inflammation. Various *in vivo* studies are being carried out to show recruitment of DCs to areas of inflammation [33,34]. Our previous intravital imaging studies have shown that the immature DCs were maximally recruited to the neurovascular interface during EAE [8]. Now, with *ex vivo in situ* imaging, we can track these cells within the CNS of diseased animals during neuroinflammation. The EAE model used here is known to exhibit maximum leukocyte activation between days 12 and 30 post inoculation, with sclerotic lesions being known to induce the generation and release of CCL2 [35,36]. Anti-CD3 reactive T cells as well as macrophages are reported to cluster just outside the blood vessels within superficial white matter along with demyelinating lesions [25], while peripheral macrophages are accepted as the primary targets of CCL2-directed chemotaxis [37]. Near-infrared and *ex vivo* microscopic images from studies presented here clearly indicate the recruitment of DCs from the perivasculature into areas of EAE, strengthening the case that DCs also transmigrate to the brain from the systemic circulation. Further, DCs were found in close juxtaposition with T cells and areas of MBP lesions, illustrating the potential function of DCs in antigen presentation to T cells (Figure 1B,C). We were also able to implicate CCL2 as an important inflammatory mediator involved in this recruitment emanating from the region with ongoing demyelination (Figure 2) and from *in vitro* transmigration assays.

A closer look at the monolayer model of the BBB in our experiments showed that brain microvascular endothelial cells do exhibit considerable resistance (Figure 3A) as compared with vascular endothelia of other body organs that are known to have high permeability [38]. From both one-cell and three-cell BBB models we concluded that DCs bear the maximum transmigratory potential in their native state as compared with naïve CD4 or CD8 T cells (Figures 4A and 5A). Also, these cells responded maximally to the presence of CCL2 in both nonactivated and activated form (Figures 4B and 5B). We further took into account the dual role that CCL2 plays in affecting behavior and functionality of immune cells and altering the physiology of endothelial cells. CCL2 has been shown to breach the BBB by binding to CCR2 receptors on the endothelium, thereby altering the expression of tight junction proteins [20,39]. Attenuation in caveolin-1 levels have also been shown to be a result of loss of BBB integrity [40]. In accordance, we saw a decrease in the expression of ICAM-1 and caveolin-1 as well as gap formation in the endothelial monolayer following treatment with CCL2 (Figure 6A). The transwell imaging results corroborated the transmigration assays allowing visualization of the kinetics of DC migration over a 24-hour period (Figure 6A). Further, these analyses revealed for the first time distinct transmigratory paths that DCs and T cells utilize, with DC movement being paracellular (Figure 6B; Movie S1 in Additional file 1) and T cells moving primarily transcellularly (Figure 6B; Movie S2 in Additional file 2) as was also shown previously [41]. From these results, we concluded that CCL2-mediated alteration of the BBB structure may facilitate paracellular passage of DCs – in that increased gap formation between endothelial cells during active inflammation may allow DCs to squeeze through easily.

In order to dissect molecular pathways involved in the migratory pattern of DCs versus T cells, we examined the role of two well-characterized signaling molecules (p38-MAPK and ERK1/2) based on the existing literature on cellular trafficking. Interestingly, in response to CCL2 we observed an ERK-dependent migratory response in DCs, whereas T-cell migration was clearly dependent on p38-MAPK signaling (Figure 7). Similar observations were made in monocytes, where p38 and ERK signaling were thought to play separate roles in cellular migration and adhesion, respectively [42]. It is possible that CCL2 stimulation of DCs leads to increased expression of receptors that facilitates tethering and adhesion to the endothelia, thus increasing the migratory potential. For example, CD49d integrin is involved in both the rolling and tight adhesion steps of extravasation, and has been shown to be an important mediator in DC recruitment [8]. The regulation of adhesion molecules is probably different in DCs and T cells in response to CCL2, which may explicate the contrast in transmigration efficiency.

In summary, these unique comprehensive studies are the first to demonstrate that CCL2 is an important mediator in the chemoattraction of DCs to the BBB. A dialogue of DC migration is significant because of their role as antigen-presenting cells in both innate and adaptive immunity, and detection of DC infiltration in the EAE model will extend the physiological relevance of investigation into the mechanistic interaction of DCs with the BBB. For example, by disrupting their ability migrate to the CNS, we can attenuate the ability of DCs to propagate the spread and relapse of EAE. In addition, CCL2 has been found to play a role in cancer [43], angiogenesis [44], bone remodeling [45], and HIV encephalitis [46], in which a common underlying pathogenic factor is CCL2-driven immune cell recruitment. These studies lay the groundwork for a new understanding of CCL2 in neuroinflammation and autoimmunity, extending beyond the classically defined role in immune cell recruitment to include DCs. Mechanistically, following the multistep leukocyte cascade paradigm, DCs may use specific cellular adhesion molecules to tether along the endothelia, become stimulated by chemokines, and begin its maturation process, leading to an upregulation of integrins involved in firm adhesion to and eventual transmigration across the BBB. A recent study of monocyte recruitment and activation at the site of the BBB and eventual maturation in the brain supports the notion that a similar mechanism may influence DC recruitment [47].

## Abbreviations

BBB: Blood–brain barrier; CCL2: Chemokine ligand 2; CNS: Central nervous system; DAPI: 4',6-diamidino-2-phenylindole; DC: Dendritic cell; EAE: Experimental autoimmune encephalomyelitis; ERK: Extracellular related kinase; MAPK: Mitogen-activated protein kinase; MBP: Myelin basic protein; MDDC: Monocyte-derived dendritic cell; MFI: Mean fluorescence intensity; PBL: Peripheral blood lymphocyte; PBS: Phosphate-buffered saline; TEER: Trans-endothelial electrical resistance.

## Competing interests

The authors declare that they have no competing interests.

## Authors’ contributions

Conception and design (DS, AL, CAF, PJ). Acquisition of data, (DS, AL, CAF). Analysis and interpretation of data (DS, AL, CAF, PJ). Drafting the manuscript (DS, AL, CAF, PJ). Revising it critically for important intellectual content (CAF, MGP, ZKK, PJ). Final approval of the version to be published (MGP, ZKK, PJ). All authors read and approved the final manuscript.

## Supplementary Material

Additional file 1 **Movie S1.** Paracellular transmigration of MDDCs. Primary DAPI-labeled MDDCs added to the upper chamber of the transwell. After 30 min, cells were stained for ICAM-1 (green) or caveolin-1 (red), viewed at 100× magnification and Z-stacked to obtain a series ofimages revealing position of MDDCs migrating relative to endothelial cellmarkers. Click here for file

Additional file 2 **Movie S2.** Paracellular transmigration of MDDCs. Primary DAPI-labeled MDDCs added to the upper chamber of the transwell. After 30 min, cells were stained for ICAM-1 (green) or caveolin-1 (red), viewed at 100× magnification and Z-stacked to obtain a series ofimages revealing position of MDDCs migrating relative to endothelial cellmarkers. Click here for file
